# Ethnobotanical study on plants used for traditional beekeeping by Dulong people in Yunnan, China

**DOI:** 10.1186/s13002-020-00414-z

**Published:** 2020-10-14

**Authors:** Zhuo Cheng, Binsheng Luo, Qiong Fang, Chunlin Long

**Affiliations:** 1 College of Life and Environmental Sciences, Minzu University of China, Beijing, 100081 China; 2 Key Laboratory of Ethnomedicine (Minzu University of China), Ministry of Education, Beijing, 100081 China; 3 Kunming Institute of Botany, Chinese Academy of Sciences, Kunming, 650201 China

**Keywords:** *Apis cerana cerana*, Biodiversity conservation, Dulong ethnic group, Ecosystem services, Traditional beekeeping, Traditional ecological knowledge

## Abstract

**Background:**

The Dulong (Drung) people have used plant materials in traditional beekeeping for many decades. However, there are few studies on the plants used in traditional beekeeping. Furthermore, traditional ecological knowledge (TEK) associated with beekeeping is still poorly understood. TEK and plants associated with beekeeping play an important role in the conservation of native bees and the development of beekeeping. It is therefore very urgent to investigate, record, and study the plants and TEK of Dulong beekeeping.

**Methods:**

Fieldwork was conducted in the Dulong community of Gongshan County, Yunnan Province, China. Six Dulong villages were investigated. Ethnobotanical methods such as free listing, semi-structured interviews, participatory observation, and key informant interviews were used to collect data. A total of 42 Dulong respondents provided information about plants used in traditional beekeeping. TEK related to traditional beekeeping plants was documented. Citation frequency, abundance, and preference ranking of log beehive plant species were used to identify plant resources that are “easier to obtain” and “more preferred.”

**Results:**

There are two general methods of traditional Dulong beekeeping: living tree beekeeping and log beehive beekeeping. The investigation revealed that 38 species (in 19 families), including 30 tree species, 5 bamboo species, 2 herbaceous species, and 1 liana species, are used in traditional Dulong beekeeping. Different plant parts are used for different purposes. Twenty-seven tree species are used to make log beehives. Species from the family Pinaceae and Fagaceae are the most frequently represented. Seven of the most commonly reported species used to build log beehives were scored by ten beekeepers. Based on this scoring, the beekeepers’ most preferred species for making log beehives are *Alnus nepalensis*, *Pinus yunnanensis*, and *Juglans regia*.

**Conclusion:**

The Dulong people have used various plants for traditional beekeeping and have accumulated rich TEK associated with apiculture. Future research will include a nutritive components analysis of honey from traditional Dulong beekeeping and an ethnobotanical investigation of melliferous species used in traditional Dulong apicultural systems. The application of plants and TEK associated with beekeeping is important for improving livelihoods in local communities, conserving biocultural diversity, and protecting the eco-environment of the Dulongjiang area.

## Background

Traditional beekeeping is important for agriculture, rural employment, human nutrition, and economic development. It has become an example of successful livelihood improvement in remote area of developing countries [1–3]. Traditional beekeeping has many advantages in that it carries low risk, requires little investment, generates active income, and saves labor [4, 5]. At the household level, beekeeping can provide nutritious products, such as honey, beeswax, royal jelly, and propolis. Selling these bee products can help poor farmers transition out of poverty [6, 7]. In crop fields, bees are important pollinators that improve yields and promote sustainable development [8, 9]. At the ecosystem level, beekeeping plays an indispensable role in protecting biodiversity and maintaining ecological balance [10–12].

Traditional beekeeping has been practicing as a conventional occupation in China’s vast rural areas for several thousand years [13]. China has high honeybee diversity, a long history of beekeeping, and many managed honeybee colonies [14]. For a long time, China has been among the world’s leaders in number of bee colonies, annual honey production, and export of bee products [15]. Traditional beekeeping has made a significant contribution to Chinese beekeeping [16]. Collecting honey in tree holes can be traced back 2000 years. In the book “Golden Rules of Business Success” (*Zhi Fu Qi Shu* in Chinese) written by Fan Li during the Spring and Autumn Period (ca.770 to 476 BC), there are sections describing traditional beekeeping with a wooden box placed in a courtyard [14]. Traditionally, man-made devices are mainly used for breeding Chinese bees (*Apis cerana cerana*) and most man-made devices belong to ethnic minorities in the mountainous areas of Southwest China. Many studies have reported that traditional beekeeping can protect native bees [17–21].

Dulong (or Drung), one of China’s less populous ethnic groups, are concentrated in the Dulongjiang area, living mainly near water and in forests [22]. As a result of their long history of interacting with the living environment and of the unique topography, rainy weather, closed traffic conditions, and abundant natural resources of their lands, the Dulong people have developed many unique livelihood strategies, such as collecting wild edible and medicinal plants, fishing, hunting, beekeeping, and slash-and-burn agriculture [23]. In traditional Dulong beekeeping practice, different plants have been used by the beekeepers in different ways, especially in the construction of log beehives and shelters for *A*. *cerana*. However, it is uncertain what kinds of log beehive materials the Dulong prefer and how they sustainably manage these plant resources. Research in other regions has shown that traditional ecological knowledge (TEK) and plants associated with beekeeping are important for sustainable development of beekeeping and for ecosystem stability. Strengthening the management of these plants can contribute to biodiversity conservation and ecosystem services [17, 21, 24].

Traditional beekeeping has become the main income source for Dulong people. Unfortunately, little has been reported about the plants used in traditional Dulong beekeeping or about the related TEK. With the development of tourism in the Dulongjiang area, TEK of beekeeping will inevitably be affected. Due to China’s rapid economic development, traditional modes of production and the associated traditional culture has been changing dramatically in the Dulong community. It is very urgent to investigate, record, and study the plants, processes, and traditional ecological knowledge used in Dulong traditional beekeeping. This study aimed to (1) document the process of traditional Dulong beekeeping and associated TEK, (2) report plants used in traditional Dulong beekeeping, (3) evaluate plant species that are “easier to obtain” and “more preferred” for log beehive materials, and (4) analyze the benefits and challenges of traditional Dulong beekeeping.

## Methods

### Study area

**Fig. 1:**
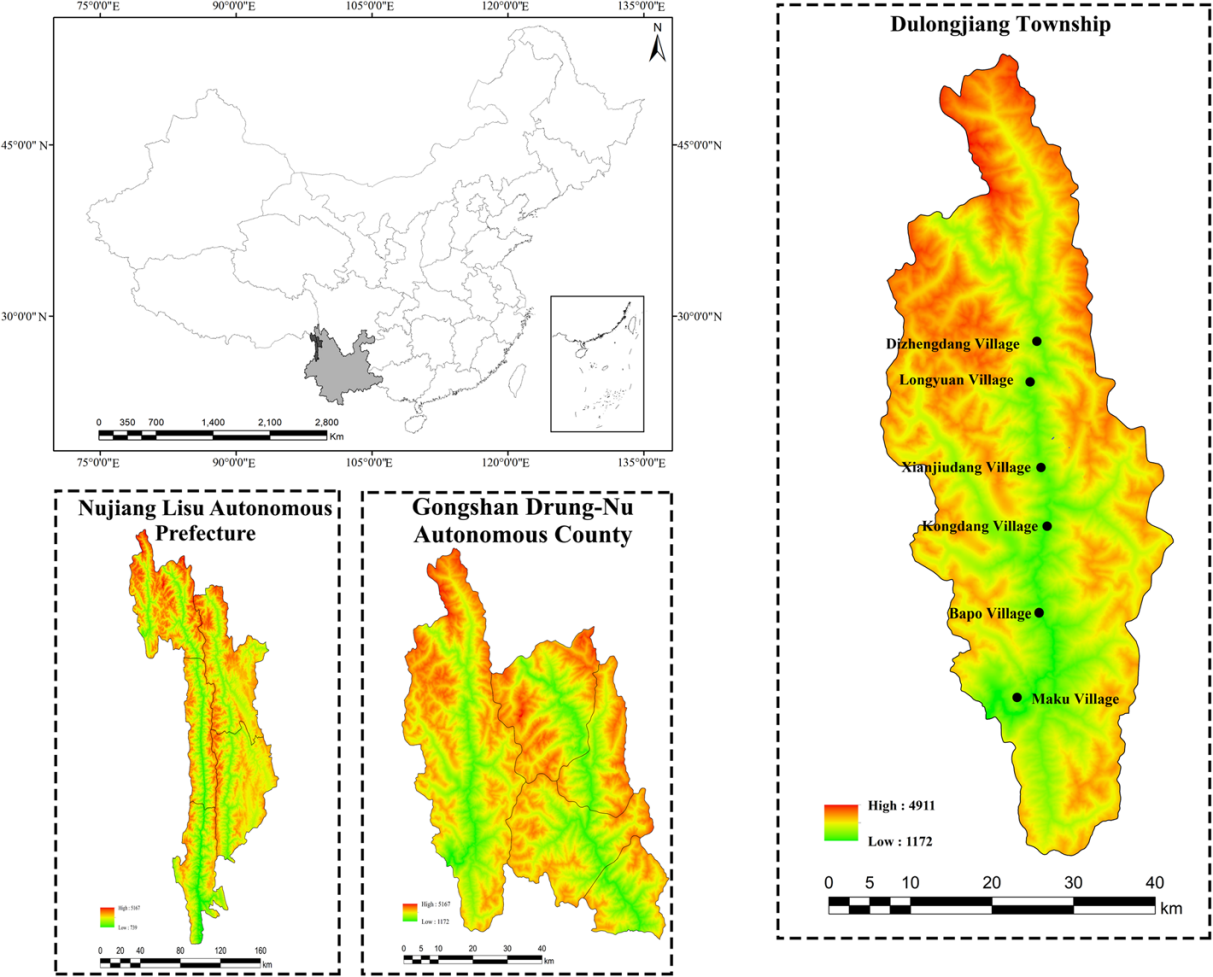
Study sites

Dulongjiang Township, the only settlement of the Dulong (Drung) people in China, is located in the west of Gongshan Dulong and Nu Autonomous County, Nujiang Lisu Autonomous Prefecture, Yunnan Province, China (Fig. 1). Dulongjiang Township (27° 40′ ~ 28° 50′ N, 97° 45′ ~ 98° 30′ E) borders Bingzhongluo and Cikai townships to the east, Chayu County of Tibet Autonomous Region to the north, and Kachin State of the Federation of Myanmar to the west and south [25]. Dulongjiang Township has a typical alpine-gorge landscape with a large altitude gradient ranging from 222 to 3400 meters above sea level. The average rainfall is 3672.8 mm per year. Dulongjiang Township is one of the core areas of the Gaoligongshan National Nature Reserve and the World Natural Heritage of Three Rivers Parallel. It is one of the highest biodiversity areas of China, with 275 species in 41 families of ferns and 2003 species in 158 families of seed plants [25–28].

Dulong is one of the 55 ethnic groups in China and is the smallest ethnic group by population in Yunnan Province. The 6930 Dulong people living in Dulongjiang Township account for 99% of the total population. Most people speak the Dulong language. Only young people can speak Mandarin Chinese. These indigenous people have minimal income, most of which is generated from plantations of spices and herbs [29–31].

### Field survey and data collection

**Fig. 2:**
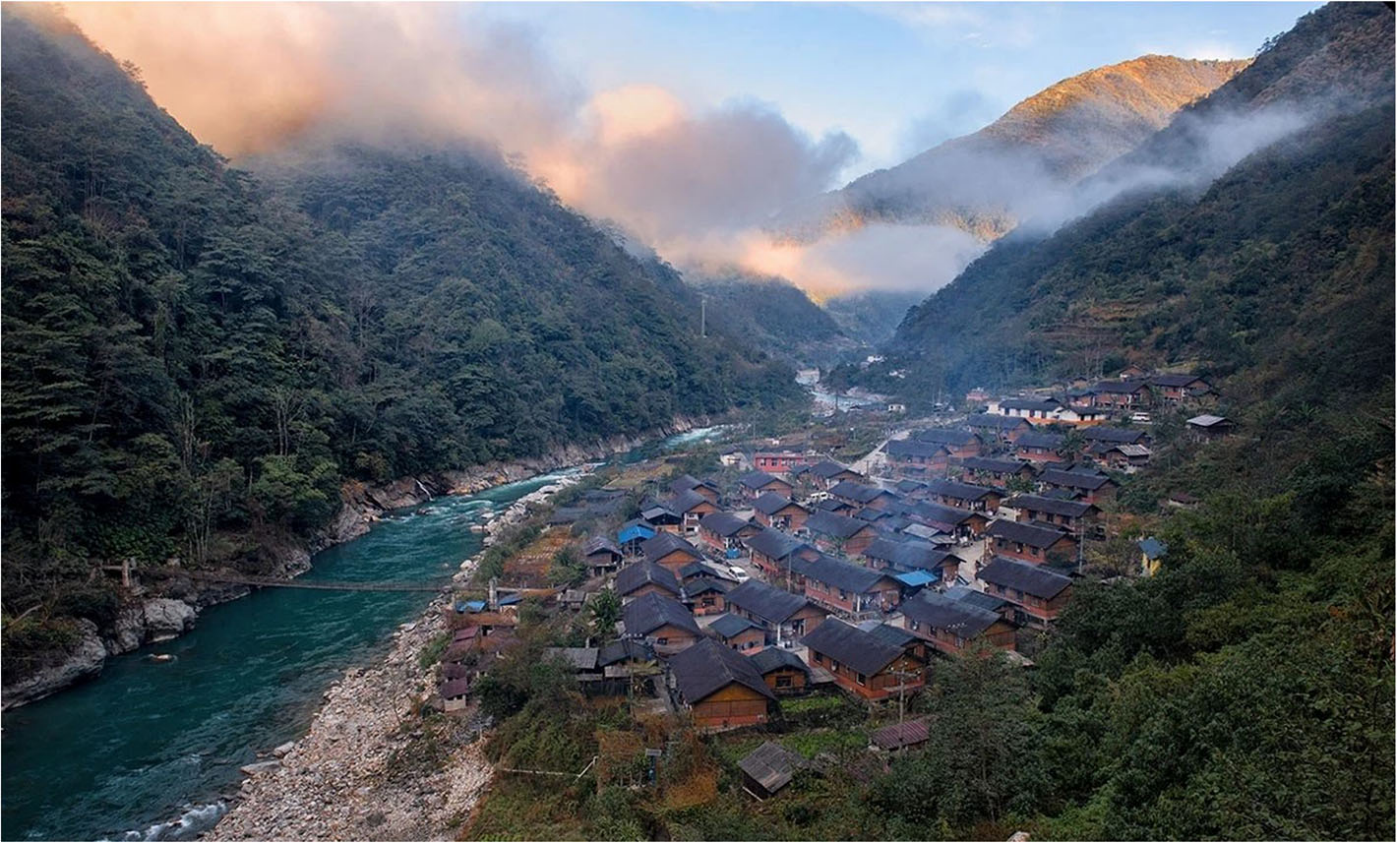
Landscape of Bapo Village

During August and November 2019, we conducted ethnobotanical surveys in Dulongjiang Township covering villages of Xiongdang, Dizhengdang, Longyuan, Xianjiudang, Kongdang, Bapo, and Miaku (Fig. 1, Fig. 2).

Ethnobotanical methods including free listing, semi-structured interviews, participatory investigation, and key informant interviews were used in the field investigations. A total of 42 informants were selected using snowball sampling. In the first stage, participants were invited to freely list all plants used in beekeeping. Then, semi-structured interviews were conducted. Interviews (Table 1) were conducted in Mandarin Chinese and translated into Dulong language with the assistance of our Dulong guide. Informed consent was obtained verbally from all participants prior to the study. After obtaining permission, photos were taken of the main steps in the beekeeping process.

**Table 1:** Questions used for semi-structured interviews

1	What plants do you usually use in beekeeping?
2	What are the local names of the plants used for beekeeping?
3	Where do you collect the plants used for beekeeping?
4	Are there a lot of resources for this plant species?
5	Which log beehive materials do you think is better for beekeeping?
6	Based on what principles do you choose log beehive materials?
7	What are the threats to beekeeping?
8	How can we conserve the native bee species?

In the second stage of field surveys, researchers accompanied by local villagers conducted a field walk to collect plant species used in beekeeping and to observe the beehives. Some plant species used to make log beehives were not found during the field survey. Pictures from the *Plant Photo Bank of China* (http://ppbc.iplant.cn/) of these plants were shown to informants to confirm species identification and other relevant information. The nomenclature of all vascular plants reported in our study follows the *Flora of China* [32]. Prof. Chunlin Long identified the plant species, and the voucher specimens were deposited at the herbarium in College of Life and Environmental Sciences, Minzu University of China, in Beijing.

### Demographic characteristics of the respondents

A total of 42 Dulong respondents (7 from Bapo, 8 from Kongdang, 8 from Xianjiudang, 6 from Longyuan, 7 from Maku, and 6 from Dizhengdang) were selected for interviews. The study found that activities related to traditional beekeeping are generally performed by men; thirty-eight of the respondents were male, and only four were female. The respondents’ ages ranged from 25 to 73 years old, and the average age was 41. Most beekeepers were farmers. The overall level of education was low. Young people had higher levels of education than the elders (Table 2).

**Table 2:** Demographic characteristics of respondents

Characteristics		Frequency
Formal education		
	Illiterate	8
	Primary school	10
	Middle high school	18
	High school	6
Main occupation		
	Farming	30
	Salary work	11
	Trading	1
Gender		
	Male	38
	Female	4

### Quantitative analysis

To identify plant resources used for log beehive materials that are “easier to obtain” and “more popular,” we used questions 1, 4, and 5 in Table 1 to determine citation frequency (how many times the informants mentioned each plant), abundance, and preference ranking (PR) of log beehive plants [33]. Frequency of citation was the key factor in prioritizing log beehive materials. Abundance of log beehive species can represent the resource and accessibility of plants, and frequency of citation was calculated based on the work of Zhang et al. [34]. Preference ranking was used with ten selected informants—regarded as professional beekeepers in their village—to rank the most popular log beehive plant species in the study area. All informants were asked to score the seven plant species with the highest citation frequency, with a score of 5 meaning highly preferred and a score of 1 meaning highly undesirable [35, 36].

## Results and discussions

### The process of traditional Dulong beekeeping

**Fig. 3:**
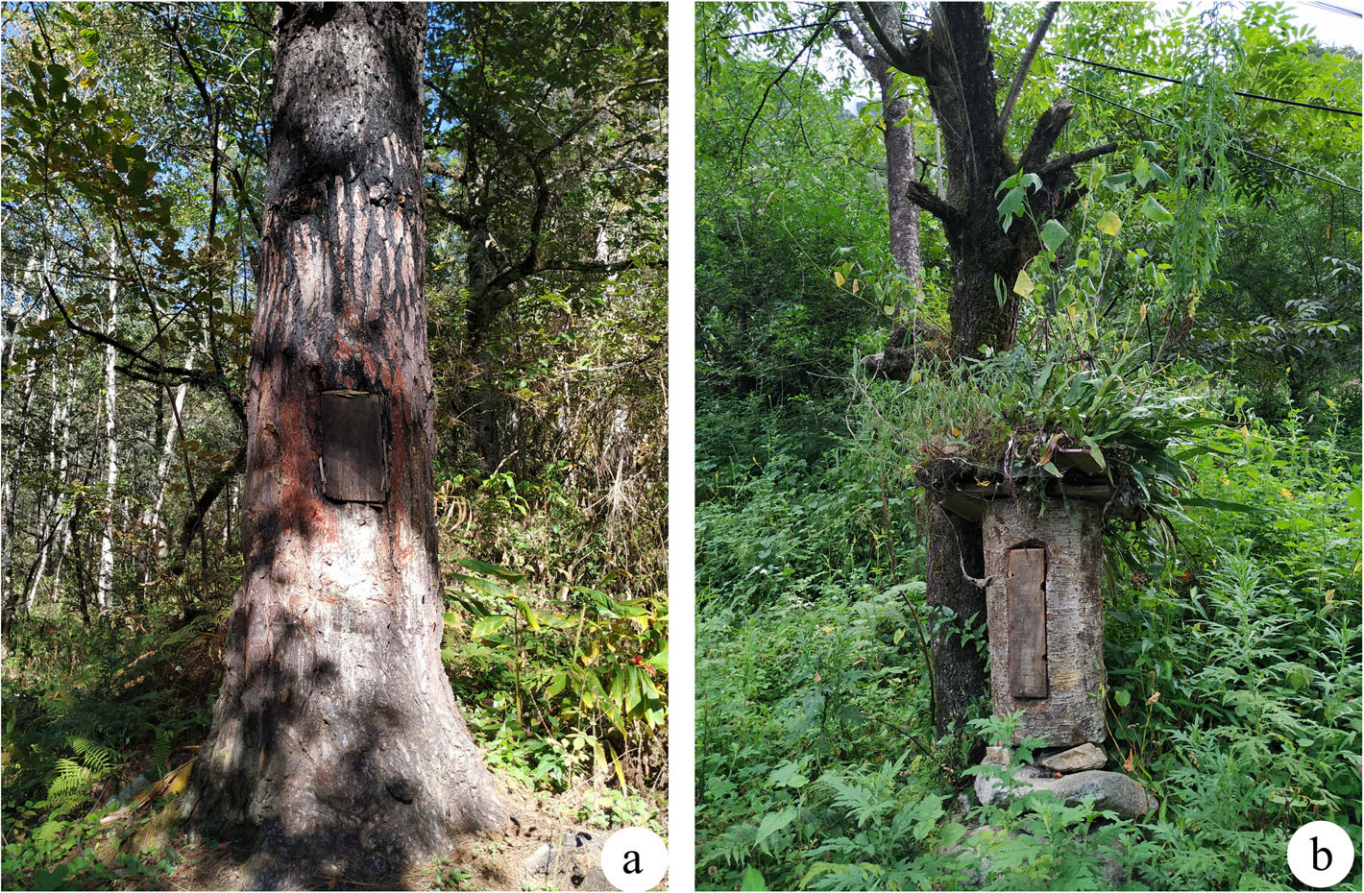
Two general forms of traditional Dulong beekeeping. **a** Living tree beekeeping. **b** Log beehive beekeeping

There are two general forms of traditional Dulong beekeeping in the study area: living tree beekeeping and log beehive beekeeping (Fig. 3a, b). Living tree beekeeping is relatively rare because of the complexity of the process, which requires more time and effort to construct the beehives. Generally, it is only done in the trunks of *Pinus yunnanensis* and *Alnus nepalensis*. The Dulong people use a tree with holes or chisel a rectangular hole about 1.5 m above the ground, cover the nest door, and leave a few small holes next to it. They then burn the inside of beehives with fire, and finally coat the inside with mud. The advantage of living tree beekeeping is that it stimulates the natural environment and avoids destruction by wild animals such as black bear (*Ursus thibetanus*).

**Fig. 4:**
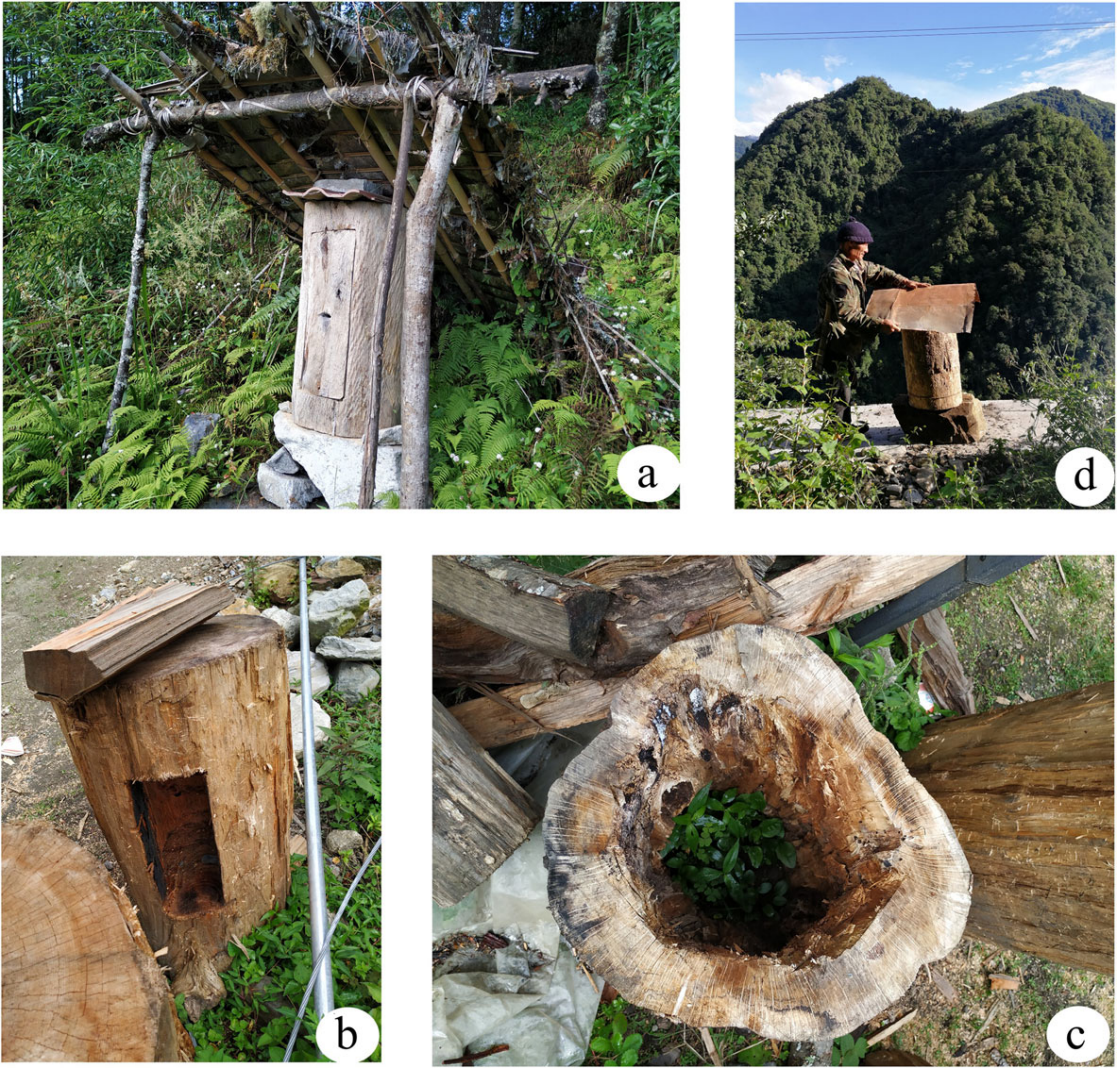
The process of making log beehives and shelters. **a** Completed log beehives and shelters. **b** Constructing log beehives. **c** Applying various pre-treatments. **d** Installing log beehives

The process of log beehive beekeeping generally includes construction, applying various pre-treatments, and installation (Fig. 4b–d). Log beehives are mostly made of round wood 70 to 100 cm in length and 30 to 50 cm in diameter. They are made from dead trunks or wood salvaged from the river.

There are two ways to make nest doors to log beehives: hollowed from the side or hollowed on both ends. If the log is hollowed from the side (Fig. 4b), the hole is covered with a 20 cm × 10 cm rectangular wooden board. If the log is hollowed from both ends (Fig. 4c), one end is sealed with a circular wooden board whose diameter is close to that of the hole. There are various ways of constructing the nest door on the other end of the log to allow bees to move in and out of the hive. For example, holes can be opened up on the nest door, or a small gap can be left between the nest door and the log, which allows 4–5 bees at a time to pass through. After the preparation, various pre-treatments, such as drying and removing worms from the log beehive, are required. Some beekeepers bake the inside of log beehives with fire. Others use chopsticks to form a cross shape inside the beehive, making it easier to cut honey.

**Fig. 5:**
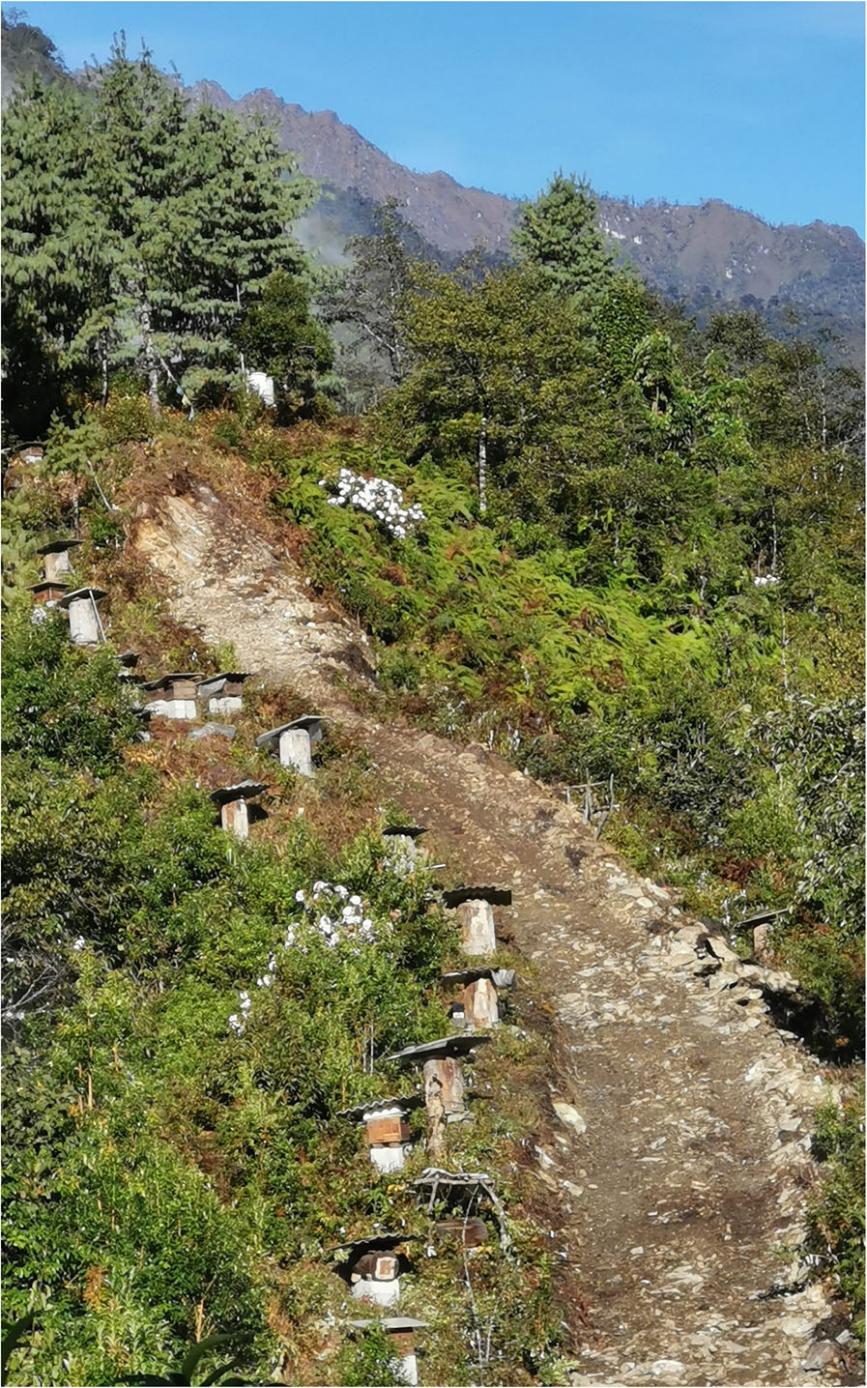
View of numerous log beehives

Most beekeepers install their hives on the leeward side of a structure. Log beehives are placed behind houses or on cliffs and cannot be reached from the ground. To prevent the log beehives from getting wet, the Dulong build a shelter made of bamboo and rattan, cover the hive with leaves of *Caryota obtusa* and *Magnolia rostrata*, or put a wooden board on top of the log beehives (Fig. 4a). With modernization, more artificial materials, such as rope and iron plates, are being used in log beehive construction. However, these modern materials are not as effective as the traditional materials. To prevent damage from wild animals and natural disasters, beekeepers use natural barriers to shield the hives. They also concentrate beehives on cliffs, which forms a beautiful landscape (Fig. 5).

**Table 3:** Plants used in traditional beekeeping by the Dulong people

Family	Scientific name	Vernacular name	Citation frequency	Abundance	Part(s) used	Use(s)	Voucher number
Actinidiaceae	*Saurauia napaulensis* DC.	da bu qiu	**	****	Stem	Log beehive	DXB0013
Actinidiaceae	*Saurauia griffithii* Dyer	da bu rong	**	***	Stem	Log beehive	DXB0012
Anacardiaceae	*Toxicodendron vernicifluum* (Stokes) F.A. Barkley	de k ri	*****	***	Stem	Log beehive	DXB0014
Arecaceae	*Caryota obtusa* Griff	a lei	**	*	Leaf	Shelter	DXB0005
Berberidaceae	*Holboellia angustifolia* Wall.	–	*	**	Bark	Affixing hive to base	DXB0044
Betulaceae	*Alnus nepalensis* D. Don	si mer	*****	*****	Stem	Log beehive	DXB0002
Betulaceae	*Betula alnoides* Buch.-Ham. ex D. Don	da m kei	***	**	Stem	Log beehive	DXB0056
Cupressaceae	*Taiwania cryptomerioides* Hayata	–	*	**	Stem	Log beehive	DXB0018
Cupressaceae	*Platycladus orientalis* (L.) Franco	su ba	*	**	Stem	Log beehive	DXB0015
Elaeocarpaceae	*Elaeocarpus borealiyunnanensis* H.T. Chang	me li	**	*	Stem	Log beehive	DXB0023
Fagaceae	*Castanea mollissima* Blume	bang li	***	***	Stem	Log beehive	DXB0054
Fagaceae	*Quercus glauca* Thunb.	si na	*****	*****	Stem	Log beehive	DXB0024
Fagaceae	*Quercus lamellosa* Sm.	si sai	*	*	Stem	Log beehive	DXB0010
Fagaceae	*Lithocarpus dealbatus* (Hook. f. and Thomson ex Miq.) Rehder	leng	*	*	Stem	Log beehive	DXB0008
Fagaceae	*Lithocarpus hancei* (Benth.) Rehder	leng	*	*	Stem	Log beehive	DXB0009
Juglandaceae	*Juglans mandshurica* Maxim	me li bu	*	*	Stem	Log beehive	DXB0057
Juglandaceae	*Juglans regia* L.	bu	*****	*****	Stem	Log beehive	DXB0055
Lauraceae	*Cinnamomum camphora* (L.) J. Presl	chun	*****	***	Stem	Log beehive	DXB0004
Lauraceae	*Machilus yunnanensis* Lecomte	–	*	***	Stem	Log beehive	DXB0007
Magnoliaceae	*Magnolia rostrata* W.W.Sm.	houpu	**	*	Leaf	Shelter	DXB0006
Meliaceae	*Toona sinensis* (Juss.) M. Roem.	zong	*****	***	Stem	Log beehive	DXB0043
Pinaceae	*Abies delavayi* Franch	zi se	*	*	Stem	Log beehive	DXB0046
Pinaceae	*Picea brachytyla* var. *complanata* (Mast.) W.C. Cheng ex Rehder	dang	*	*	Stem	Log beehive	DXB0047
Pinaceae	*Pinus armandii* Franch.	dang	***	***	Stem	Log beehive	DXB0022
Pinaceae	*Pinus wallichiana* A.B. Jacks.	gui ning	*	***	Stem	Log beehive	DXB0028
Pinaceae	*Pinus yunnanensis* Franch.	dang me	*****	****	Stem	Log beehive	DXB0026
Pinaceae	*Tsuga dumosa* (D.Don) Eichler	–	*	*	Stem	Log beehive	–
Poaceae	*Chimonobambusa armata* (Gamble) Hsueh and T.P. Yi	gu er	*	***	Stem, Bark	Shelter, Affixing hive to base	DXB0063
Poaceae	*Sinocalamus fugongensis* (Hsueh and D.Z. Li) W.T. Lin	de wa	*	****	Stem	Shelter	DXB0065
Poaceae	*Fargesia pleniculmis* (Hand.-Mazz.) T.P. Yi	de ma	*	**	Stem, Bark	Shelter, Affixing hive to base	DXB0046
Poaceae	*Fargesia praecipua* T.P. Yi	si wen	*	**	Stem Bark	Shelter, Affixing hive to base	DXB0045
Poaceae	*Phyllostachys sulphurea* (Carrière) Rivière and C. Rivière	xing na gan	*	****	Stem Bark	Shelter, Affixing hive to base	DXB0042
Poaceae	*Imperata cylindrica* (L.) Raeusch.	–	*	***	Whole plant	Shelter, Drive swarm away	DXB0061
Rubiaceae	*Luculia yunnanensis* S. Y. Hu	long gang	**	*	Flower	Swarm attraction	DXB0065
Salicaceae	*Populus szechuanica* C.K. Schneid.	–	**	**	Stem	Log beehive	DXB0020
Sapindaceae	*Acer oliverianum* Pax	–	***	**	Stem	Log beehive	–
Taxaceae	*Taxus wallichiana* Zucc.	qiang deng	*	*	Stem	Log beehive	–
Urticaceae	*Urtica laetevirens* Maxim.	–	*	**	Bark	Affixing hive to base	DXB0058

Species in this inventory are listed alphabetically, first by family and then by scientific name. Vernacular names of plants are written in Chinese pinyin

*Citation frequency and abundance

### Plant species used in traditional Dulong beekeeping

**Fig. 6:**
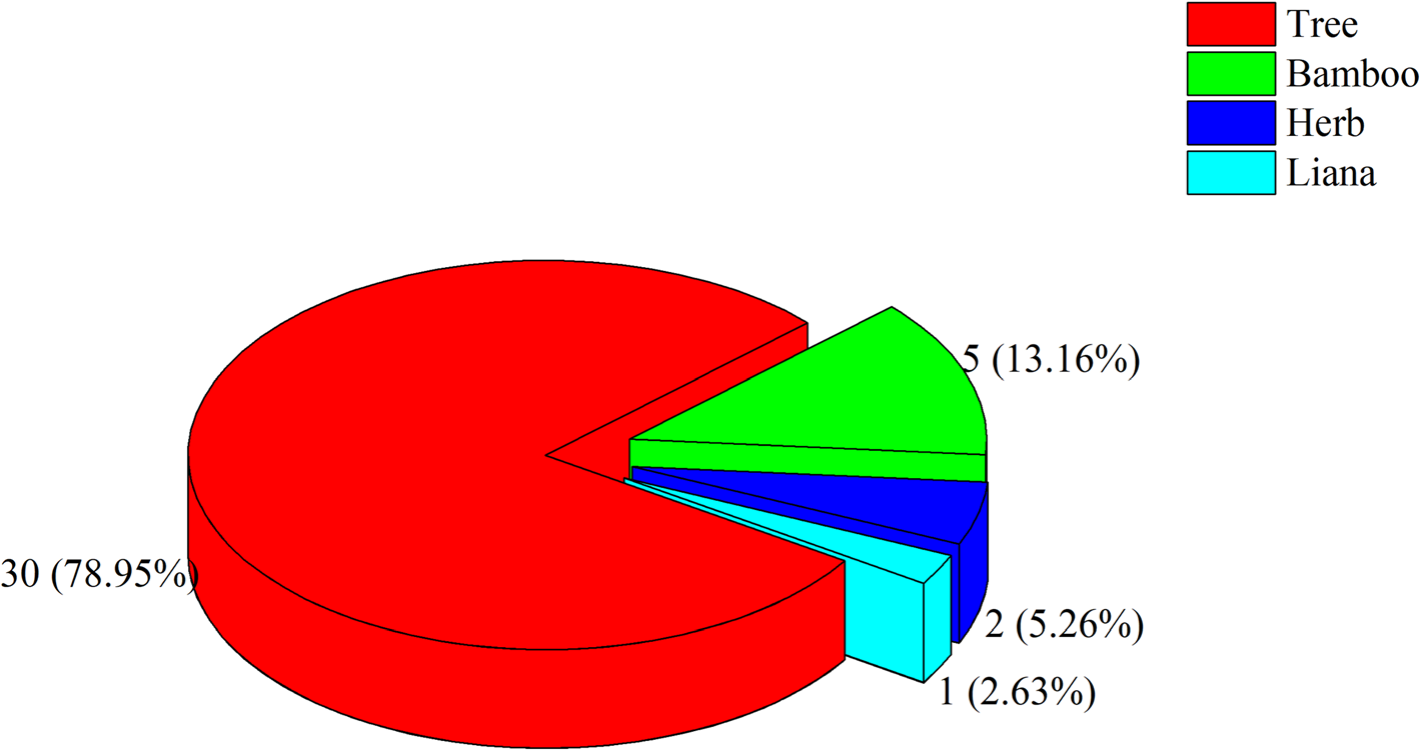
Life form of plants used in traditional Dulong beekeeping

Thirty-eight species—including 30 tree species—belonging to 19 plant families were documented through interviews of 42 informants from six villages (Fig. 6) (Table 3). Different plant parts were reported by beekeepers for constructing shelters and log beehives, for driving swarms away during honey collection, for affixing hives to their bases, and for attracting swarms. All tree species, except *Magnolia officinalis*, *Caryota obtusa*, and *Luculia yunnanensis*, are used for making log beehives. Leaves of *Magnolia officinalis* and *Caryota obtusa* are used for shelter construction, and flowers of *Luculia yunnanensis* are used to attract swarms. All bamboo species are used to make shelters, while *Chimonobambusa armata*, *Fargesia pleniculmis*, and *Fargesia praecipua* are also used for affixing hives. One herb (*Urtica laetevirens*) is processed into twine for affixing while the other (*Imperata cylindrica*) is used for making shelters and driving swarms away. Dulong people burn the whole plant of *I*. *cylindrica* when collecting honey to drive swarms away. Only one liana (*Holboellia angustifolia*) is used in traditional beekeeping, for affixing.

**Fig. 7:**
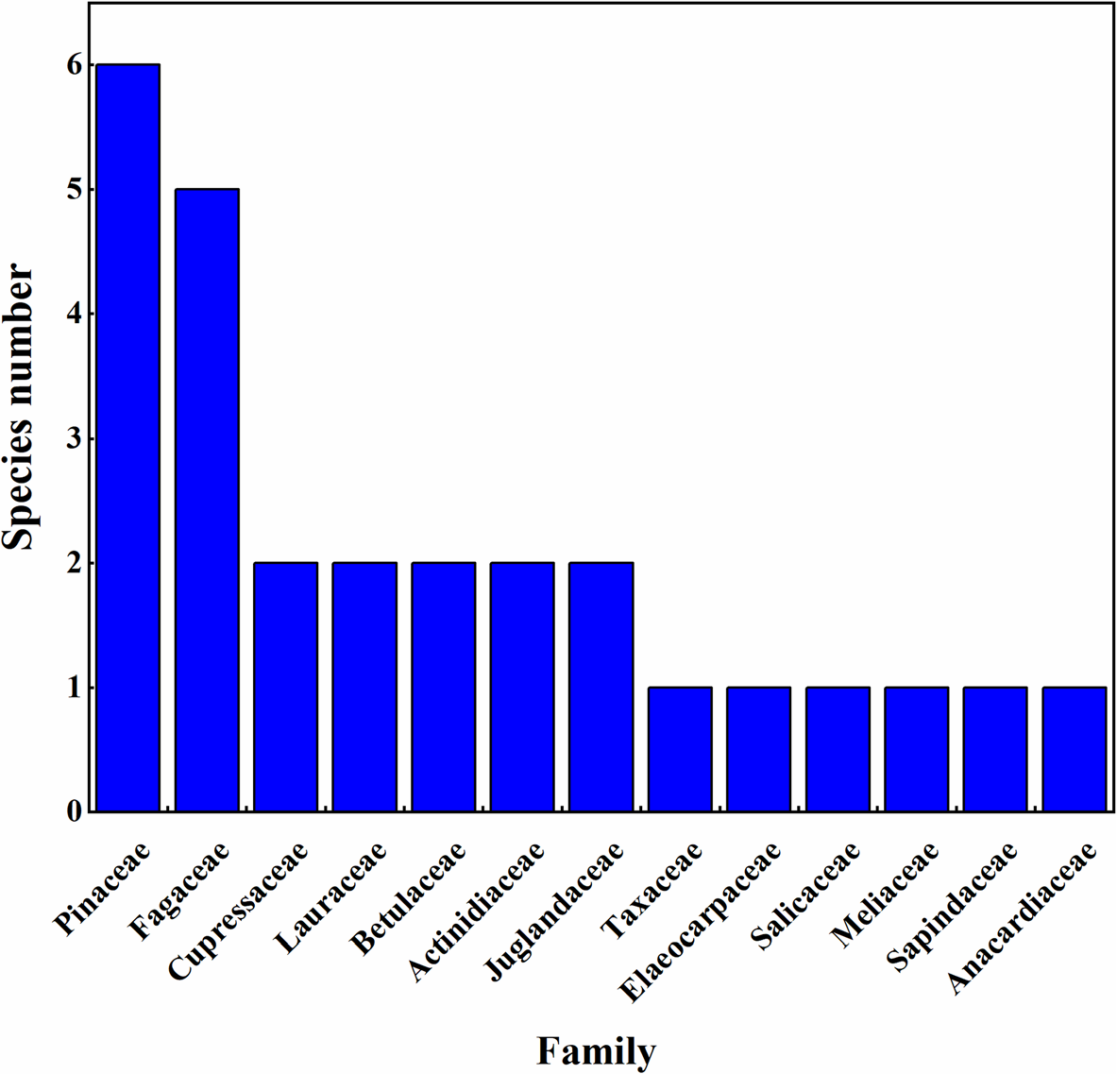
Species frequencies of botanical families used in log beehive construction

A total of 27 tree species are used for making log beehives. In terms of plant families, Pinaceae and Fagaceae have the greatest representation of species with 6 and 5 species, respectively, while other families are only represented by one or two species (Fig. 7). In our survey, seven most cited plants (mentioned by more than 75% of respondents) were *Pinus yunnanensis*, *Cinnamomum camphora*, *Juglans regia*, *Alnus nepalensis*, *Quercus lamellosa*, *Toxicodendron vernicifluum*, and *Toona sinensis*. The most abundant plants were *Pinus yunnanensis*, *Quercus lamellosa*, *Alnus nepalensis*, and *Juglans regia*.

Six plants used in beekeeping are included in the national list of protected species. For example, the trunks of *Taxus wallichiana*, *Taiwania cryptomerioide*, *Picea brachytyla* var. *complanata*, and *Cinnamomum camphora* are used to make log beehive, and the leaves of *Houpoea rostrata* and *Caryota obtusa* are used to make shelters. The Dulong do not cut down these endangered plants. The materials used for making log beehives are generally sourced from dead trunks or wood salvaged from the river, which are easy to process. This also avoids cutting living trees.

### Evaluation of tree species used for making log beehives

The survey results show that among the most commonly used materials, the beekeepers’ favorite materials are *Alnus nepalensis*, *Pinus yunnanensis*, *Juglans regia*, *Cinnamomum camphora*, *Toxicodendron vernicifluum*, *Toona sinensis*, and *Cyclobalanopsis glauca*. During the investigation, we found that beekeepers generally choose log beehive materials based on four criteria: abundance, ease of processing, durability, and smell. Different tree species have pros and cons in various aspects. For example, *Pinus yunnanensis*, *Alnus nepalensis*, *Quercus lamellosa*, and *Juglans regia* are very common and easy to obtain. In terms of durability, *Pinus yunnanensis*, *Quercus glauca*, and *Cinnamomum camphora* are the best, because they are excellent materials for buildings. However, materials that are more durable are also more challenging to process. *Alnus nepalensis*, *Juglans regia*, and *Toona sinensis* are easier to process. In terms of smell, *Toxicodendron vernicifluum*, *Toona sinensis*, and *Cinnamomum camphora* have strong odors while the odor of *Pinus yunnanensis* is faint. Some beekeepers prefer constructing bee barrels from plants with strong odors because the odor discourages pests and disease, while others believe that strong odors are bad because bees avoid or dislike the smell. Based on abundance, ease of processing, durability, and smell, the tree species most highly valued by beekeepers for log beehives are *Alnus nepalensis*, *Pinus yunnanensis*, and *Juglans regia* (Table 4).

**Table 4:** Preference ranking of the seven beehive plant species with the highest citation frequency, based on scoring by ten beekeepers

Respondent	*Pinus yunnanensis*	*Cinnamomum camphora*	*Cyclobalanopsis glauca*	*Juglans regia*	*Alnus nepalensis*	*Toxicodendron vernicifluum*	*Toona sinensis*
R1	1	1	1	4	4	5	5
R2	3	1	1	4	5	1	1
R3	4	1	1	4	5	1	1
R4	5	2	1	3	4	1	1
R5	1	3	1	5	5	3	2
R6	4	1	3	4	4	1	1
R7	5	1	4	3	3	1	1
R8	5	4	1	1	2	4	4
R9	5	4	1	1	1	4	4
R10	1	2	1	5	5	1	1
Total	34	20	15	34	38	22	21
Rank	2nd	6th	7th	2rd	1st	4th	5th

*R* respondent

### Benefits and challenges of traditional Dulong beekeeping

**Fig. 8:**
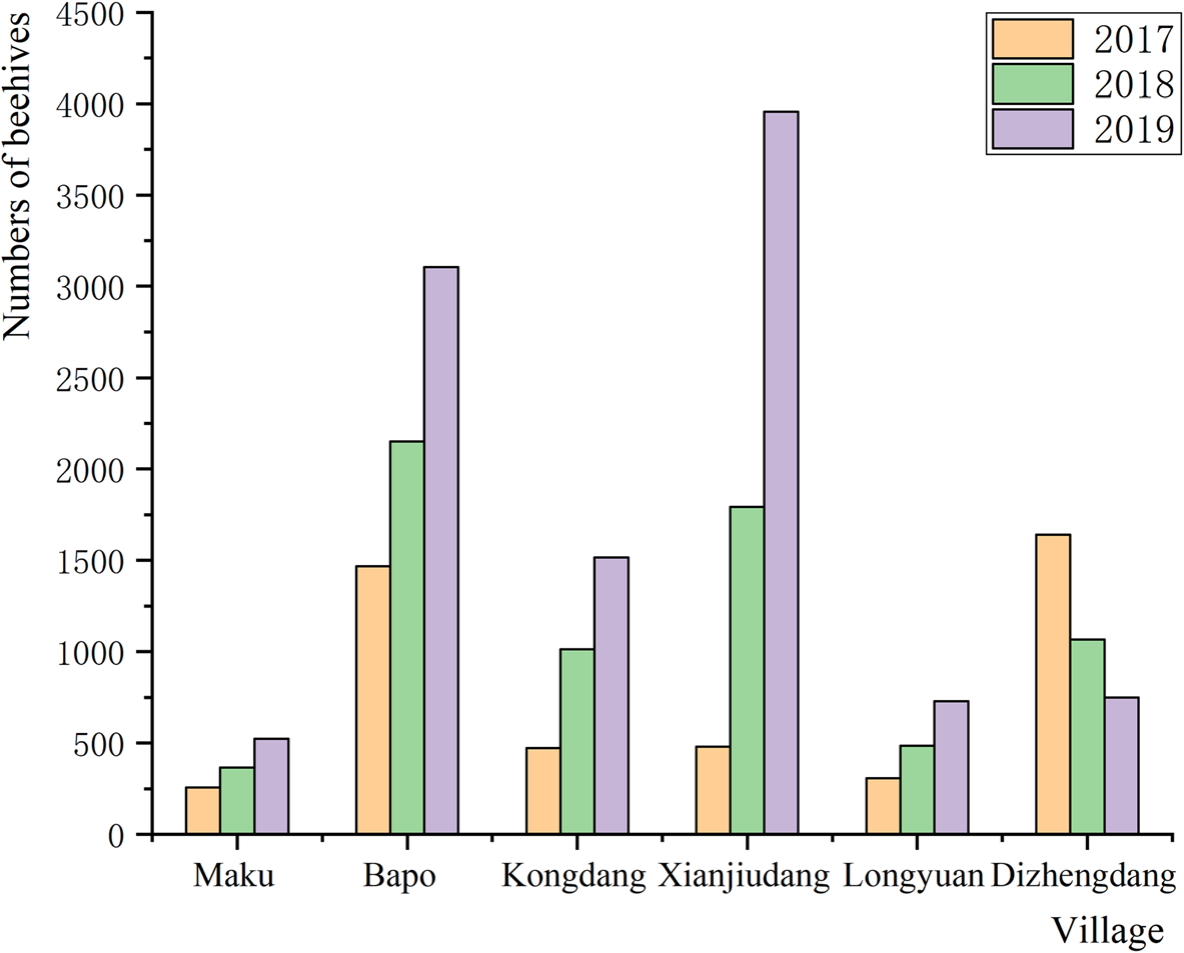
The number of beehives in Dulongjiang Township (2017–2019)

**Fig. 9:**
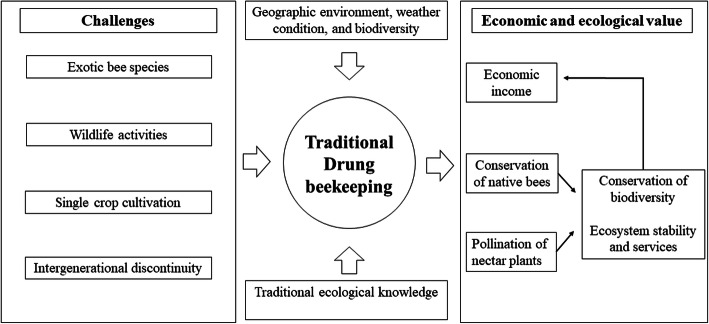
Benefits and challenges of traditional Dulong beekeeping

Dulong people use traditional beekeeping methods to keep native bees (*A*. *cerana*). The scale of traditional Dulong beekeeping in Dulongjiang area continues to grow. Except for Dizhengdang Village, the number of beehives in the other five villages has been increasing in the past three years (Fig. 8). The unique geography, weather conditions, and rich biodiversity of the Dulongjiang area provide abundant nectariferous plants for native bees, which provides a solid ecological basis for maintaining traditional beekeeping. In addition, the unique beekeeping methods and rich TEK provide a better chance of survival for native bees (Fig. 9). There has been a growing interest in the development of bee products and their medical, health, diet, and beauty benefits, but little attention has been paid to the crucial role of beekeeping in maintaining ecological balance. Traditional beekeeping is the main income of the Dulong, and it also protects the native bee species and promotes the pollination of nectariferous plants. In addition, it plays an irreplaceable role in maintaining ecosystem stability and promoting ecosystem services of the Dulongjiang area. Over time, traditional beekeeping promotes a sustainable, eco-agricultural economy while increasing income for local residents (Fig. 9).

#### Conservation of native bees and pollination of nectar plants

Traditional Dulong beekeeping has played a key role in promoting the growth of *A*. *cerana* populations, making the native bees very prosperous in the Dulongjiang area. Dulong beekeeping devices, bee colony domestication, pest control, honey cutting technologies, cultural constraints, and other factors related to traditional beekeeping support the protection of native bees and local biodiversity (Table 5). Pollination by bees is essential for nectariferous plants, providing ecosystem services and contributing to ecosystem stability at the same time [37, 38]. Native bees have formed symbiotic relationships with other species during their long-term evolution in the Dulongjiang area. It is this symbiosis that plays a vital role in maintaining local ecological equilibrium, especially for nectariferous plants that rely on *A*. *cerana* for pollination.

**Table 5:** TEK of traditional Dulong people’s beekeeping

Aspects	TEK
Beekeeping devices	The materials used for making log beehives are generally sourced from dead trunks or wood salvaged from the river, which are easy to process and avert the need to cut living trees.
Bee colony domestication	Dulong people do not introduce exotic bee species to increase honey production. They believe that exotic bee species will compete with native species in foraging, pollination, and nesting.
Pest control	Dulong people believe using fertilizers and pesticides will cause severe damage to local flora and fauna. They achieve pest control by burning the inside of beehive with fire and then coating the inner side with mud.
Honey cutting	When collecting honey, Dulong people leave half of the honey in the beehives to prevent native bees from starvation caused by lack of honey in the winter.
Nectariferous plants	Dulong people place beehives around important winter nectariferous plants like *Eurya*, *Luculia*, *Schefflera*, and *Persicaria*, which helps prevent bees from starving in extreme weather.
Cultural constraints	Dulong people regard *Platycladus orientalis* as a god tree and believe that felling it brings bad luck. Thus, *P*. *orientalis* is rarely used as beehive material. The Dulong never use *Pinus griffithii* for log beehives because they think it is an exotic species and has the potential to harm local species.

#### Contradictions between wildlife protection and traditional beekeeping

**Fig. 10:**
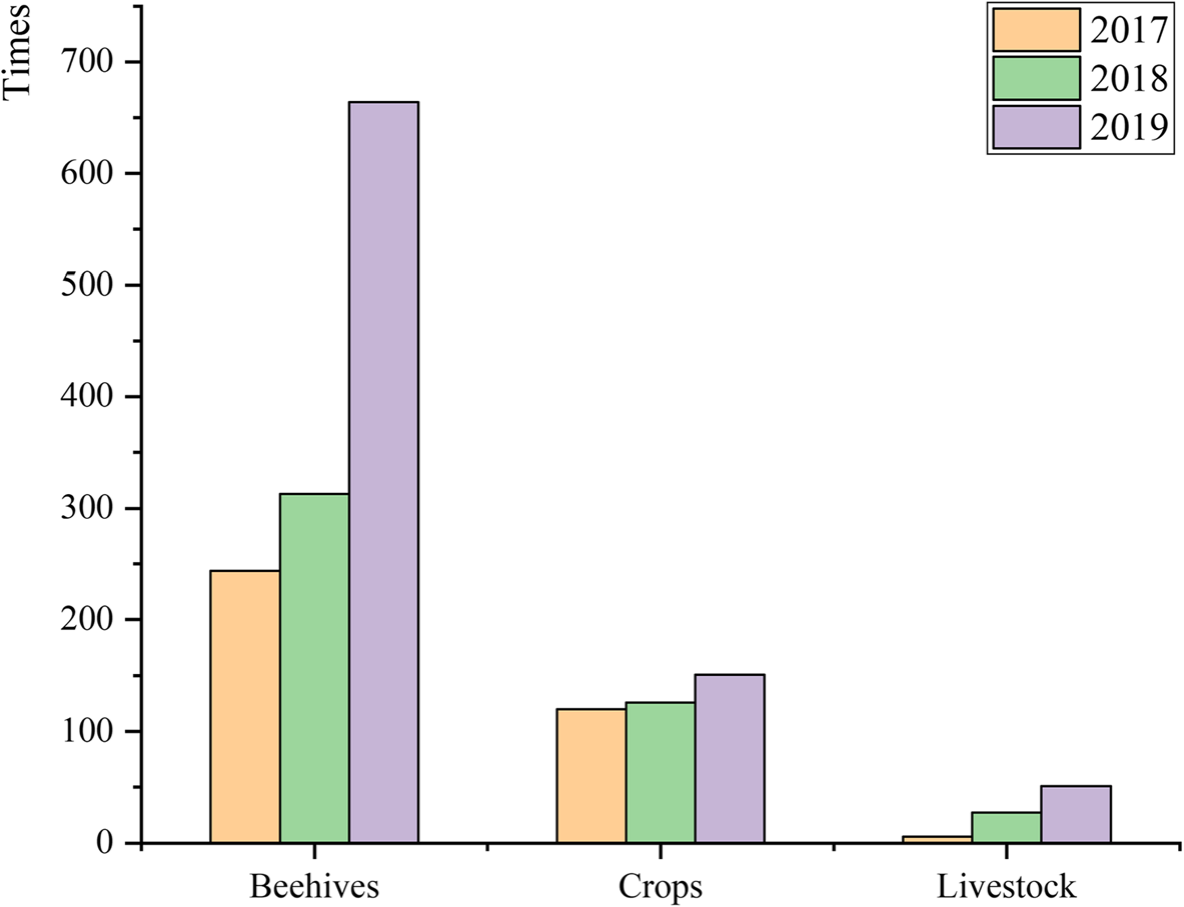
The numbers of wildlife accidents in Dulongjiang Township (2017–2019)

The Dulongjiang area has one of the highest abundances of wildlife in China. It is the core area of the Gaoligongshan National Nature Reserve, which is known as the “Animal and Plant Gene Bank” [26]. With the implementation of the natural forest protection project and reverting farmlands to forests, the number of wild animals and their activities has also increased significantly [32]. Beehives in the Dulongjiang area have always been under threat of destruction by wild animals, especially by bear (*Ursus thibetanus*). Wild animal incidents with beehives, crops, and livestock are increasing year by year in Dulongjiang Township, resulting in huge economic losses to local residents (Fig. 10).

*Ursus thibetanus* (black bear) is a protected animal species listed in the Red-data Book of China (http://www.zoology.csdb.cn/). Beekeepers are not allowed to harm these animals, reflecting an apparent contradiction between wild animal protection and traditional beekeeping. This contradiction not only causes severe losses to beekeepers’ income but also dispels local people’s enthusiasm in beekeeping.

**Fig. 11:**
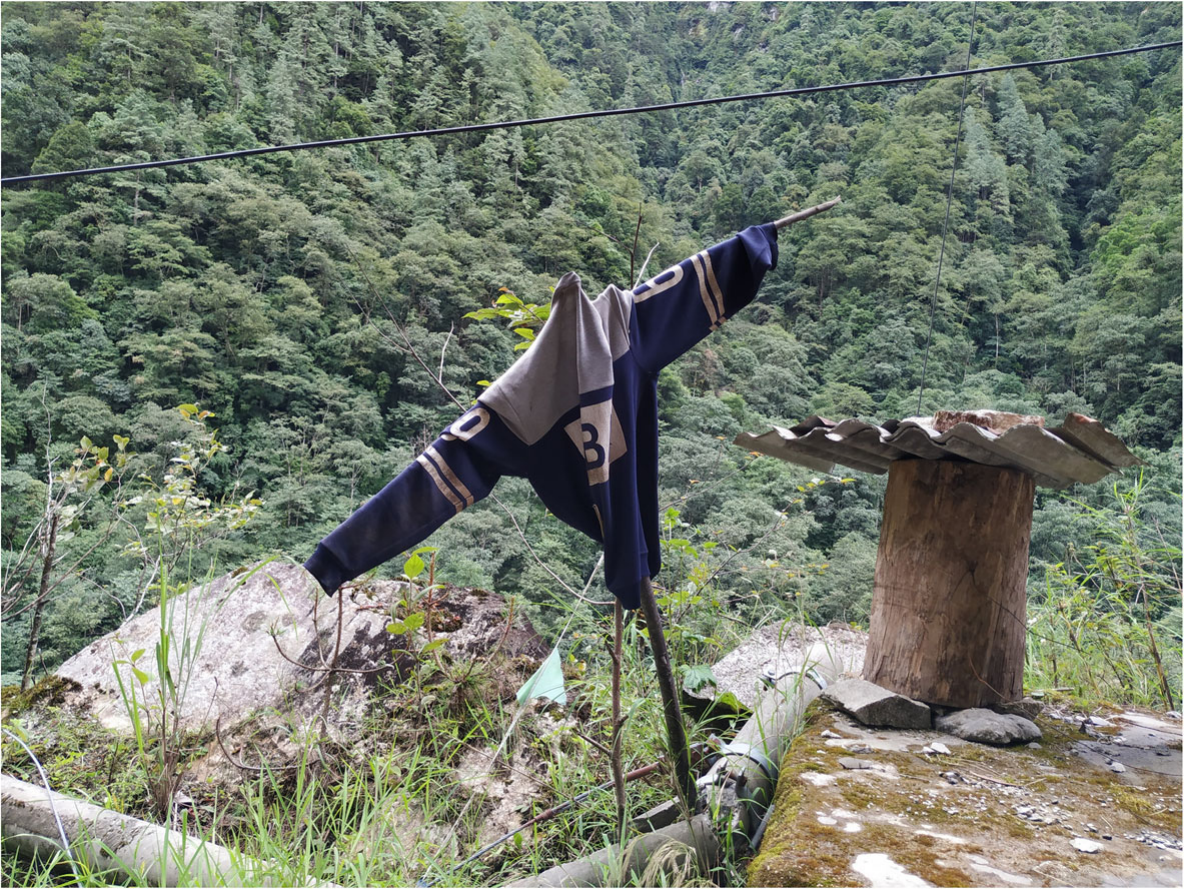
A bear-repellent device close to a log beehive

The regional protection bureau has formulated corresponding compensation measures for damages to beekeeping caused by wild animals. After the beehives are damaged, forest rangers assess the losses. The losses are then compensated for after reporting. Although this method can partially compensate for beekeepers’ losses, evidence of animal damage is hard to collect, and the process can sometimes take a long time, which also decreases beekeepers’ enthusiasm. It is suggested that the local nature protection department work together with local beekeepers to find appropriate solutions, such as beekeeping insurance and establishment of compensation measures suitable for local beekeepers, to ensure that every household who suffers a loss can be compensated properly. Traditional bear-repellent devices, such as scarecrows generally used by Dulong (Fig. 11), have achieved excellent effects in reducing losses. This effective, traditional method of repelling wild animals is also worth promoting.

#### Exotic bee species, single crop cultivation, and intergenerational discontinuity

Although the geography and weather conditions of the Dulongjiang area are not suitable for exotic bee species to survive, the danger of exotic bee species’ invasion still exists. Monitoring the behavior of non-local beekeepers entering the Dulongjiang area is strongly recommended. In recent years, the Dulong have planted large amounts of *Amomum tsaoko*, which has brought huge economic benefits. However, single crop cultivation may reduce the distribution of melliferous plants and is not conducive to the survival of native bees. With Dulong people emerging out of poverty, enormous changes have taken place in transportation and economics in the Dulongjiang area. The TEK of beekeeping will inevitably be impacted by modernization. Therefore, it is imperative to record, study, and maintain this TEK. Many local young people are reluctant to participate in beekeeping or learn traditional knowledge. They prefer to work outside or grow cash crops rather than learn the complex trade of beekeeping. The traditional knowledge of beekeeping faces the danger that no one will inherit and perpetuate this knowledge. Therefore, we suggest that training beekeepers on proper beekeeping techniques that attract young generations can promote the protection of TEK.

## Conclusion

Traditional Dulong beekeeping uses a variety of plants, and its associated TEK is rich. Thirty-eight species (in 19 families) used in beekeeping and 27 tree species used to make log beehives were recorded. Species from the family Pinaceae and Fagaceae are the most frequently represented. *Alnus nepalensis*, *Pinus yunnanensis*, and *Juglans regia* are preferred by beekeepers for making log beehive because they are abundant, easy to process, durable, and have desirable aromas. Future work will include analysis of nutritive components of honey from traditional Dulong beekeeping and ethnobotanical investigation of melliferous species used in traditional Dulong beekeeping will be conducted. It is necessary to take full advantage of traditional beekeeping to promote a sustainable economy, protect biodiversity, and improve the livelihood of local communities. Thus, a win-win situation can be achieved for culture, ecology, and economy.

## Data Availability

All data generated or analyzed during this study are included in this published article.
